# Examining Patterns of Information Exchange and Social Support in a Web-Based Health Community: Exponential Random Graph Models

**DOI:** 10.2196/18062

**Published:** 2020-09-29

**Authors:** Xuan Liu, Shan Jiang, Min Sun, Xiaotong Chi

**Affiliations:** 1 East China University of Science and Technology Shanghai China; 2 University of Massachusetts Boston Boston, MA United States

**Keywords:** web-based health communities, information exchange, social support, ERGM

## Abstract

**Background:**

Although an increasing number of studies have attempted to understand how people interact with others in web-based health communities, studies focusing on understanding individuals’ patterns of information exchange and social support in web-based health communities are still limited. In this paper, we discuss how patients’ social interactions develop into social networks based on a network exchange framework and empirically validate the framework in web-based health care community contexts.

**Objective:**

This study aims to explore various patterns of information exchange and social support in web-based health care communities and identify factors that affect such patterns.

**Methods:**

Using social network analysis and text mining techniques, we empirically validated a network exchange framework on a 10-year data set collected from a popular web-based health community. A reply network was extracted from the data set, and exponential random graph models were used to discover patterns of information exchange and social support from the network.

**Results:**

Results showed that reciprocated information exchange was common in web-based health communities. The homophily effect existed in general conversations but was weakened when exchanging knowledge. New members in web-based health communities tended to receive more support. Furthermore, polarized sentiment increases the chances of receiving replies, and optimistic users play an important role in providing social support to the entire community.

**Conclusions:**

This study complements the literature on network exchange theories and contributes to a better understanding of social exchange patterns in the web-based health care context. Practically, this study can help web-based patients obtain information and social support more effectively.

## Introduction

### Background

The rapid evolution of the internet and related technologies has created advanced virtual platforms that allow popular and pressing health topics to be discussed on the web. Unlike in the past, patients now may seek help from experts, share stories with similar patients from across the world to obtain emotional support, and keep themselves informed with latest updates about related issues [1]. The resulting environment is often referred to as web-based health communities, where users can share relevant experiences about diseases, physical conditions, and therapeutic schedules. Users can also consult specialists and seek opinions from experts [2]. Various web-based health communities such as PatientsLikeMe, CureTogether, DailyStrength, and Disaboom are emerging. In these platforms, patients can read stories shared by other peers, find information regarding diseases, post a new thread to initiate a conversation, and reply to others’ messages to provide feedback. During these interactions, information exchange and social support occur [3]. Users can provide information by leaving messages, which generates a dynamic information exchange procedure [4]. Meanwhile, obtaining social support, especially emotional relief, from communicating with others has become one of the major purposes for patients with diseases to join web-based communities [5]. An increasing number of studies have attempted to understand how individuals interact with others in web-based health communities. For example, research has shown that patients with similar disease stages or health status are more likely to develop friendship on the web [6]. Urban users tend to provide social support to rural participants in web-based health communities [7]. A recent study has found that web-based interaction between doctors and patients does not decrease the effectiveness of information exchange compared with face-to-face communication [8]. However, studies focusing on understanding individuals’ patterns of information exchange and social support in web-based health communities are still limited.

The aim of our research is to explore various patterns of information exchange and social support in web-based health care communities and identify factors that affect such patterns. On the basis of the network exchange framework [9], we discuss how patients’ social interactions develop into social networks. We empirically validated the network exchange framework based on data sets collected from a leading web-based health community in China. The results from our analyses indicate that reciprocated information exchanges are likely to develop between patients, especially between web-based members who have different roles and members who are web-based friends. Some patients are more likely to receive social support, especially when they are new to the community and when they express polarized sentiment in messages. In addition, these patterns could vary depending on the topics being discussed by patients. To the best of our knowledge, this study is the first to validate the network exchange framework in the web-based health community context. This study is also the first to perform a stratified analysis of user sentiment to understand the complex information exchange and social support patterns between patients with various sentiments.

### Related Work

#### Information Exchange and Social Support

In web-based health communities, patients can exchange information by sharing their experiences in overcoming illness, transferring medical knowledge to one another, and providing information regarding health care resources. Different communities usually specialize in different aspects. Some communities focus on specific types of disease, whereas others can provide unique services to patients. For example, in DailyStrength, patients with anxiety disorder can find a support group suggested by the community to discuss how to overcome stress or other disorders. In Tianmijiayuan, patients with diabetes and their families can post messages to share how they deal with different stages of diabetes in the long term. PatientsLikeMe provides a matching service for patients based on their profiles to quickly get in touch with other peers who have experienced or are experiencing similar diseases. Members of web-based health communities can benefit from their collective knowledge and skills by exchanging information among each other [10]. Information exchange is also a critical component in the development of many web-based communities [11]. Information exchange in web-based health communities can enhance effective communication between medical experts and patients by improving teamwork [12].

Previous studies have shown that social support plays a crucial role in helping individuals improve their health status or treat psychological problems [13,14]. The benefits of web-based social support come in 2 forms: informational support and emotional support [15].

By asking questions regarding health concerns on the web, users can obtain professional knowledge from experts. Moreover, patients may learn experiences from others who share similar diseases. Although such informational benefits can also come from information exchanges, obtaining social support differs from information exchange mainly in the patterns of interaction. Unlike information exchanges where the interaction is mutual, social support can be unilateral [16]. Many users provide informational support without expecting any return due to empathy [17].

Emotional support mainly comes from web-based users who share similar disease experiences or from friends of such patients. Patients can talk about what difficulties they have overcome, what they did to recover, and can encourage peers to be optimistic and fight against the disease. Some patients have reported that they received more understanding from web-based strangers than they did from offline families or friends [18]. This support could significantly enhance patients’ emotional well-being [19]. As such, web-based health communities have become a platform for many patients to seek and provide emotional support [20].

Previous research has implied that social network is one of the key antecedents of social support and information exchange [15]. However, understanding information exchanges and social support in web-based health communities from a social network perspective has received limited research attention.

#### Network Perspective of Web-Based Health Communities

Members of web-based health communities develop a social network through communication. Such social networks provide users an opportunity to exchange information and seek social support in web-based communities [15,21]. By using social network analysis, attributes of nodes (ie, users) and relationships between users can be modeled and examined.

Recently, a network exchange framework has been proposed to theorize how social interactions between individuals aggregate into a social network [9]. The network exchange framework tries to explain social exchange from a network perspective by combining the social exchange theory and the network theory. Social interactions are viewed as processes of exchanging resources such as information, knowledge, and emotional well-being. Individual characteristics play an important role in such exchange processes. When the pair of individuals is part of a larger network, the exchange processes can be further influenced by their positional configurations, such as their social connections with others [22]. The network exchange framework has been used to explain the formation of information exchange networks in web-based communities organized around various topics, such as software [9] and automobile [23]. However, we do not find studies that apply a network exchange framework to investigate information exchange patterns in web-based health communities. This endeavor is important because web-based health communities are distinct from other more traditional web-based communities in that information being exchanged is usually sensitive, private, and requires professional medical knowledge. Handling such information gives rise to special community norms that could lead to unique social exchange patterns. As evidenced by previous studies, patterns of social interactions could vary greatly across web-based communities in different contexts [9,24].

### Network Exchange Framework

In this study, we develop our research hypotheses regarding information exchange in web-based health communities based on a network exchange framework for several reasons. First, we deem information and knowledge exchanged between patients as resources in web-based health communities, and thus, social exchange theory can help explain the patterns of information exchange. Second, information exchange between individuals aggregates into a network between patients because patients typically interact with multiple peers. Therefore, adopting a network perspective would better explain information exchange and social support between individual patients.

In our model, we include 3 major structural tendencies that comprise network formation in the network exchange framework: direct reciprocity, indirect reciprocity, and preferential attachment [9].

#### Direct Reciprocity

According to the reciprocity principle [25], individuals expect to receive information back after providing information to others. In web-based health communities, obtaining useful health care knowledge is regarded as one of the major objectives when users join a community [26]. As individuals’ health care needs are usually complicated, users may ask further questions to obtain more information after receiving initial responses. Reciprocated information exchange develops in this way. Some users enjoy contributing their knowledge and receive thankful responses from others. Users are also likely to provide assistance to others who have provided them with support and then realize their intrinsic motivation [27,28]. Therefore, we propose the following hypothesis:

- Hypothesis 1: Reciprocated information exchange is likely to develop in web-based health communities.

The pattern of direct reciprocity could further manifest in subgroups of web-based community members. Social interaction helps people with similar characteristics to become acquainted and build trust with each other [29,30]. Homophily, the tendency for individuals to be attracted by others with similar characteristics, is an important dimension in social networks [31]. Homophily commonly occurs based on geographic and demographic characteristics such as race, religion, age, gender, residence, marital status, and interests [32,33]. In a web-based environment, similarity between users increases the frequency of their interactions [34]. A study reported that patients with similar health conditions and treatments are likely to develop friendships [6]. Another common user-related attribute in web-based health communities is user type, such as doctor, family, or patient, which users report when they join the community. As we expect homophily to exist in web-based health information exchange, we propose the following hypothesis:

- Hypothesis 2: Reciprocated information exchange is likely to develop between users who share similar concerns in web-based health communities.

Typically, web-based health community members can also become web-based friends so that they can send private messages or keep updated about each other’s posts. The possibility of sending private or offline messages may decrease their visible web-based conversations. However, being capable of following friends’ posts may increase their chances of reading friends’ messages and initiating conversations. Furthermore, web-based friendship could develop into relational capital [35], which becomes the basis for information exchange to occur. We propose the following hypothesis for empirical tests to understand the combined effects:

- Hypothesis 3: Reciprocated information exchange is likely to develop between users who are web-based friends.

#### Indirect Reciprocity

Indirect reciprocity refers to returning an information exchange but not to the original provider [36]. Indirect reciprocity can be observed in web-based health communities because communicating health information usually requires specialized knowledge, but the expertise of patients is uneven. When a patient receives informational help from a knowledge provider, the patient may not be able to return the favor due to limitations in expertise. Instead, the patient may choose to provide help to others in the network as they could feel that the help is from the community as a whole, and they are willing to return the favor to the same community [37]. Previous studies have also found that new participants who received help tend to remain in the community to help others [38]. Therefore, we propose the following hypothesis:

- Hypothesis 4: Patients who receive social support tend to provide support to others who are not necessarily the support provider.

#### Preferential Attachment

Preferential attachment refers to a process in which a new node tends to establish connections with existing nodes that already possess many connections [39]. In the context of web-based health communities, preferential attachment translates to the tendency that a patient who is already involved in many web-based social interactions is likely to receive further replies. This is intuitive because highly active members contribute more and influence more people in need [40]. Such contributions are visible to the entire community, and as a rewarding mechanism, the active members may receive more help in terms of incoming social support in the future. Therefore, we propose the following hypothesis:

- Hypothesis 5: Highly active users are more likely to receive replies as social support.

Another dimension of preferential attachment in the web-based health community context is connections with new members joining the community. Contrary to traditional preferential attachment notions, we do not expect new members to be able to select which nodes to form attachments with. This is because most new patients join a community to be helped, not to help, at least in the initial periods [26]. It is the subsequent replies to the new patient that initiate social support. In web-based health care communities, such subsequent replies are likely to occur. Although the new member has not yet contributed to the community, existing members can benefit from providing support to the new member insofar as the new member becomes part of the community and adds value through social capital to the community network [35]. As such, we expect that existing community members have greater motivation to provide social support to new members.

- Hypothesis 6: In web-based health communities, new patients are more likely to receive replies as social support.

Another factor that could affect preferential attachment in web-based health communities is sentiment. Previous studies have found that users with polarized sentiment tend to receive more attention on the web. For example, expressing positive emotions helps peers improve psychological and physical health conditions [41,42]. In web-based health communities, patients may feel more comfortable getting in touch with peers who are optimistic and show positive emotions. Negative emotions attract attention in another way. In web-based health communities, many individuals are inclined to help others avoid negative feelings, such as shame, guilt, or indebtedness, especially after they receive help from others [43]. Patients expressing negative emotions are often those who have a disease or are experiencing loss, and are in need of help from peers. Therefore, social support could also go toward patients with negative moods. Overall, we expect that users who express polarized sentiment (either positive or negative) are more likely to receive attention and hence receive more replies in web-based health communities.

- Hypothesis 7: In web-based health communities, patients with polarized sentiment are more likely to receive replies as social support.

A related question is who is providing social support to the users with polarized sentiment. On the one hand, homophily plays an important role in social networking [6,32,33], and we expect that patients with overall similar sentiment valence are likely to make friends and talk to each other very often. On the other hand, the web-based health community is a platform where patients not only make friends but also help other strangers voluntarily [5]. Without being friends or knowing someone, a patient who has gone through the most difficult time could be willing to help someone who is still suffering. Meanwhile, patients in a negative mood may seek emotional support from peers who seem to be optimistic. Therefore, in addition to the homophily effect, we also expect that users with opposite sentiments are likely to leave replies to each other. The following set of hypotheses is proposed:

- Hypothesis 8a: Patients are likely to receive replies from peers with similar sentiment valence.
- Hypothesis 8b: Patients are likely to receive replies from peers with opposite sentiment valence.

## Methods

### Data

To test our hypotheses, we collected data from Tianmijiayuan [44], a leading web-based diabetes community in China where patients, doctors, and relatives participate in various activities. It was established in 2005 and had 247,638 members in 2018. It is one of the largest and the most active web-based nonprofit Chinese diabetes communities, targeting individuals with diabetes and helping them share information about diabetes, exchange experiences of diabetes treatment, seek emotional support, and make friends with people who are facing similar diabetic conditions. From the entire forum, we extracted users’ postreply networks as well as all the textual posting content and publicly available personal information of users, such as user type and web-based friendships. Data collection was performed using a Java web crawler, with a time range from 2005 to 2015.

Tianmijiayuan has separate subforums for different discussion topics. The most popular (in terms of the number of postings) ones include *Diabetes Knowledge*, *Communications Area for Diabetics*, and *Diabetic’s Life*. The discussions in Diabetes Knowledge are usually related to symptoms and diagnoses of different types of diabetes, patients’ diet and exercise, and diabetes news. Users can make friends and participate in community activities in the Communications Area for Diabetics. In addition, they can publish their own photography, life insights, and advice for the community in the Diabetic’s Life subforum. To examine whether the information exchange and social support patterns vary depending on the topic of discussion, we also performed a separate analysis on each subforum.

### Operationalization of Nodal Attributes

The following nodal attributes were modeled in our study.

#### Individual Type

Upon registration, users choose the type of their identity as one of the following: doctors, patients’ family members, patients with type 1/2/X, web service staff, or other.

#### Activity Level

Tianmijiayuan [44] tracks a user’s number of posts, replies, web-based time, peer reviews, and numerous other factors. These factors are integrated as a numerical score to represent users’ level of activity. Users with higher scores are considered active users. We collected this information, and users whose scores ranked among the top 25% were coded as highly active users. For robustness tests, we changed this threshold value to 20%, 23%, 27%, and 30% to examine how this operationalization affects the results (seethe *Robustness Tests* section).

#### Registration Time

We classified users as long-time users or new users based on their registration time. The number of months since registration was calculated for each user, and users in the bottom 25% of registration length were coded as new users. For robustness tests, we changed this threshold value to 20%, 23%, 27%, and 30% to examine how this operationalization affects the results (see the *Robustness Tests* section).

#### Emotion

Sentiment analysis was performed to determine each user’s overall sentiment in the data set [45]. Specifically, a text analysis program, TextMind, was employed to assess users’ sentiments on Tianmijiayuan [44]. It can identify the frequency of words associated with different emotions when users express opinions in community discussions. TextMind has been used in previous research to analyze emotional expressions in Chinese texts [46]. On the basis of the frequency of emotion-related words expressed by users in the entire forum, we found that approximately 5% of users used more negative words than positive words. These users were identified as pessimistic users with negatively polarized sentiments. An equal number of users were identified as optimistic users who used more positive words than negative words (the top 5% users with the highest frequency of positive words were selected). The remaining users did not have extremely high proportion of positive or negative words and were identified as sentiment neutral users.

Table 1 summarizes the operationalization of the nodal attributes of the users.

**Table 1:** Operationalization of nodal attributes.

Node attribute	Type	Measuring method
User type, %	Categorical variable	1-Users with type 1 diabetes, 23.7 2-Users with type 2 diabetes, 58.3 3-Users with type X diabetes, 3.6 4-Family members, 7.0 5-Doctors, 0.7 6-Web service staff, 1.1 7-Others, 5.6
Activity level	Binary categorical variable	1-Highly active users 0-Other users
Registration time	Binary categorical variable	1-New users 0-Other users
Emotion	Categorical variable	2-Optimistic users 1-Pessimistic users 0-Neutral users

### Network Tie and Dichotomization

In this study, the extracted postreply network was used as the base network. If a user replied to another user’s thread post or reply post, a network tie was developed. The number of ties was counted as the network tie intensity.

Network dichotomization was then performed based on the threshold values of the tie intensity. According to a previous study [47], the threshold values were determined as the mean tie intensity plus one standard deviation.

### Exponential Random Graph Model

Exponential random graph model (ERGM) can simultaneously model structural relationships between nodes and the effects of nodes’ individual attributes on network formation [7,48,49]. The research hypotheses in our study involve various nodal attributes of web-based patients (eg, sentiment and activity level) and structural relationships between them (eg, receiving replies and reciprocating replies). With ERGM, all the complex interactions of these nodes, nodal attributes, and network ties can be incorporated simultaneously in the same model.

In ERGM, the observed network is represented as Y={Y_ij_}, where Y_ij_ indicates whether there is a tie between nodes i and j (Y_ij_=1) or not (Y_ij_=0). The ERGM generates random networks based on hypothesized network patterns (ie, configurations) and compares the generated network with the actual observed network. The more similar they are, the more likely the hypothesized network patterns exist in the actual network. The general mathematical formulation of the ERGM is as follows:

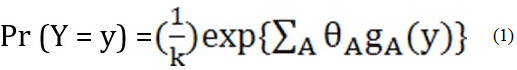



where the summation is over all configurations A, y represents one kind of particular network graph y, and 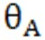 is the parameter corresponding to the configuration A. 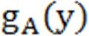 is the network statistic corresponding to configuration A, 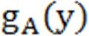
=1 if the configuration is observed in the network y and is 0 otherwise, and k is a normalizing quantity that ensures that (1) is a proper probability distribution [50]. ERGM estimates parameters 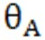 associated with each configuration, and positive and significant parameters indicate that corresponding network patterns are highly likely to occur in the network [51,52].

To test our hypotheses with ERGM, we transformed our hypotheses into network patterns. Multimedia Appendix 1 shows the hypotheses and the illustration of their network patterns.

## Results

### ERGM Results

Table 2 summarizes the estimated parameters and *P* values for all configurations. If a parameter is positive and significant, it indicates that the corresponding network pattern is more likely to develop than random chance [50]. During the initial tests, we found that the inclusion of a configuration for H4 (2-path) always resulted in model degeneracy [52]. This indicates that the pattern of indirect reciprocity hardly existed in the dichotomized postreply network. Therefore, H4 was not supported, and we excluded this network configuration from further tests.

In the subsequent section, for each hypothesis, we discuss our findings on the entire forum, and then, we compare the observations with the results in the subforums to examine how the patterns could vary depending on the topics of discussion. We deem a hypothesis to be supported only if it is supported in at least three tests.

**Table 2:** Results for exponential random graph model tests.

Configuration	Entire forum (sample size=1528)		Diabetes Knowledge (sample size=1188)			Communications Area for Diabetics (sample size=455)			Diabetic’s Life (sample size=376)		
	Coefficient	*P* value		Coefficient	*P* value		Coefficient	*P* value		Coefficient	*P* value
H1: reciprocity	3.850	<.001		3.153	<.001		3.509	<.001		3.684	<.001
H2: type	0.240	<.001		−0.139	<.001		0.188	<.001		−0.287	<.001
H3: friend	3.473	<.001		3.712	<.001		0.019	.007		0.010	.14
H5: active_user	0.012	.76		−0.220	<.001		−0.176	.002		−0.411	<.001
H6: new_user	0.409	<.001		−0.110	0.004		−0.400	<.001		−0.338	.03
H7: optimistic	0.289	<.001		−1.383	<.001		−1.050	<.001		−0.819	<.001
H7: pessimistic	0.144	.045		0.692	<.001		−2.214	.04		−0.596	.21
H8a: opti-opti	−0.332	.23		1.294	0.03		0.829	.21		−0.100	.92
H8a: pessi-pessi	−0.140	.71		−0.180	0.45		N/A^a^	<.001		N/A	<.001
H8b: opti-pessi	0.168	.51		0.281	0.17		N/A	<.001		N/A	<.001
H8b: pessi-opti	−0.218	.48		1.345	0.004		N/A	<.001		N/A	<.001

^a^N/A: not applicable.

### Hypotheses Testing Results

First, we found positive and significant coefficients for the *reciprocity* configuration in the entire forum as well as 3 popular subforums, indicating that directly reciprocated information exchange was common in web-based health communities. This observation conforms to the reciprocity principle that individuals are willing to return exchanges in favor [25,53]. In web-based health communities, patients appreciate the help received from others, and gratitude is expressed in many such reciprocated messages. In addition, we also observed that a number of patient pairs reciprocated replies in different threads, especially in the Diabetes Knowledge subforum. This indicates that patients are also willing to return favors to those from whom they have received support before. In summary, H1 was supported.

A positive and significant parameter was observed for the *type* configuration in the entire forum, indicating that users of the same type were more likely to reciprocate messages overall. However, the effect was negative and significant in the *Diabetes Knowledge* and *Diabetic’s Life* subforums. This observation implies that conversations between users of different types were more common when the discussion topics were relevant to disease knowledge (diabetes) or personal life. For example, it is very likely that diabetes patients obtain information from doctors in the *Diabetes Knowledge* subforum. Moreover, when sharing personal life with web-based peers, users may be less concerned about whether others are in the same stage of diabetes as them. Note that our finding does not imply low chances of communication between any specific pair of user types in the subforums (eg, reciprocated ties specifically between two patients with type-2 diabetes in *Diabetes Knowledge* subforum was not tested). Instead, our finding simply implies that, overall, there was more reciprocated communication between users of different types in the 2 subforums. As a result, H2 was supported in the entire forum but not in the *Diabetes Knowledge* and *Diabetic’s Life* subforums.

A positive and significant parameter was observed for the *friend* configuration in the entire forum as well as in the *Diabetes Knowledge* and *Communication* subforums, indicating that web-based friends were very likely to exchange information frequently with each other when discussing diabetes knowledge. Being web-based friends can increase one’s attention and motivation to reply to health-related posts from other community members. In addition, patients were able to obtain some timely health-related information and show empathy to others through this kind of virtual friendship [17]. This effect was not significant in *Diabetic’s Life* subforum possibly due to the fact that when sharing personal life with web-based peers, users may be less concerned about whether others are their virtual friends. Overall, H3 was supported.

The *active_user* configuration was negative and significant in all subforums. Note that the activity level of a user was evaluated based on the user’s log-in time and number of messages posted by the users in our data set. Therefore, our observation indicates that highly active users may stay on the web for a long time and leave many replies, but they may not necessarily receive an equally large number of replies back. This observation is different from previous findings that “popular friends get more friends” [6,40] but is consistent with prior research where preferential attachment was found to be in the opposite direction in knowledge sharing communities [9]. In a community where knowledge is frequently exchanged, new members do not preferably *attach* to existing active members, but instead, active members play an important role in helping new members stay in the community. In the context of our postreply network in health care communities, active members frequently help others by providing social support to them (outgoing links), but they receive relatively less support from new members (incoming links) because newcomers are usually not ready to provide help yet. Overall, H5 was not supported.

We found a positive and significant parameter estimate for the *new_user* configuration in the entire forum, indicating that new users are likely to receive replies. One of the important goals for web-based health communities is to increase community prosperity, and hence, web forums such as Tianmijiayuan [44] encourage users to help new members. Therefore, message postings from new members could be more easily noticed in the community, making the new members more likely to receive social support from other users in the web-based health community. Interestingly, this effect was negative and significant in all 3 subforums. Note that the reply networks in the subforums only counted user interactions within each subforum. Hence, our observation implies that users who recently registered tend to participate in discussions in multiple subforums rather than staying in one specific subforum. With the rapid development of internet technology, users have changed tremendously in recent years. Our results indicate that the newly joined web-based health community participants tend to utilize resources from multiple sources. Therefore, H6 was supported only in the entire forum.

Both *optimistic* and *pessimistic* configurations were positive and significant in the entire forum, indicating that patients with polarized sentiment were more likely to receive replies in the entire forum. This confirms prior findings that polarized emotion can entail more attention [41-43]. In the 3 subforums, users with polarized sentiment were less likely to receive replies in most cases, possibly because of the same reason discussed for new users. The only exception was observed in the *Diabetes Knowledge* subforum, where *the pessimistic* configuration remained positive and significant. This implies that most of the threads seeking informational support in the knowledge sharing subcommunity could be associated with negative mood. It is intuitive because patients are likely to be anxious and desperate during the information-seeking process. Moreover, giving informational support may be prioritized for patients in desperate needs due to negativity bias [54]. Overall, H7 was supported, and negative sentiment was found to have a unique impact when seeking informational support in web-based health communities.

For communication between users of similar sentiment, neither *opti-opti* nor *pessi-pessi* was significant, indicating that users with similar sentiment were exchanging messages just as normal. This observation differs slightly from findings in previous research where homophily effects manifested in more objective attributes such as gender and health status [6]. For personal attributes such as sentiment, we found that the influence effect was stronger than the homophily effect [55]. In the Diabetes Knowledge subforum, both *pessi-opti* and *opti-opti* configurations were positive and significant, indicating that optimistic users were more likely to provide support to other users who are polarized in sentiment when exchanging health care knowledge (ie, diabetes knowledge in our data set). The effect was stronger in the *pessi-opti* configuration, indicating that positive attitude can influence other users, especially those who are in a negative mood. By interacting with optimistic users, pessimistic users can obtain relief, receive encouragement, and improve emotional well-being overall. To summarize, H8a and H8b were partially supported: sentiment plays a key role in communication when information exchange is involved, and social support is more likely to come from optimistic users.

### Robustness Tests

Robustness tests conducted to examine whether operationalization of active users and new users could have affected our results. Our base experiment used a 25.00 (%) threshold to identify new users and active users. In robustness tests, we used 20.00 (%), 23.00(%), 27.00 (%), and 30.00 (%) instead to operationalize these two nodal attributes and performed ERGM analysis on the entire forum. Tables 3 and 4 show the results of the robustness tests for new users and active users. Overall, the qualitative results did not change, with the exception that the configuration for *active_user* became significant when the top 27.00 (%) or 30.00 (%) users were operationalized as highly active users. This was due to the fact that several users newly included in the robustness tests 3 and 4 posted very popular threads that received a large number of replies. Considering that the effect did not change when the threshold was changed to 20.00 (%) or 23.00 (%), we argue that being highly active did not have significant correlations with receiving support, and our qualitative results remain the same as in the base test.

**Table 3:** Results of robustness tests, new users evaluated under different thresholds.

Configuration	Exponential random graph model parameters and *P* values									
	Base test, threshold=25.00 (%)		Robustness test 1, threshold=20.00 (%)		Robustness test 2, threshold=23.00 (%)		Robustness test 3, threshold=27.00 (%)		Robustness test 4, threshold=30.00 (%)	
	Coefficient	*P* value	Coefficient	*P* value	Coefficient	*P* value	Coefficient	*P* value	Coefficient	*P* value
H1: reciprocity	3.850	<.001	4.070	<.001	4.050	<.001	3.785	<.001	3.799	<.001
H2: type	0.240	<.001	0.247	<.001	0.239	<.001	0.258	<.001	0.267	<.001
H3: friend	3.473	<.001	3.370	<.001	3.516	<.001	2.976	<.001	2.996	<.001
H5: active_user	0.012	.76	−0.005	.88	.03	.52	−0.019	.61	−0.013	.74
H6: new_user	0.409	<.001	0.487	<.001	0.432	<.001	0.362	<.001	0.316	<.001
H7: optimistic	0.289	<.001	0.305 (<.001)	<.001	0.331	<.001	0.320	<.001	0.340	<.001
H7: pessimistic	0.144	.05	0.050	.48	0.134	.08	0.118	.08	0.145	.05
H8a: opti-opti	−0.332	.23	−0.138	.64	−0.042	.89	−0.210	.43	0.117	.67
H8a: pessi-pessi	−0.140	.71	−0.244	.42	−0.432	.24	−0.319	.28	−0.757	.12
H8b: opti-pessi	0.168	.51	0.224	.38	0.200	.48	0.223	.44	0.071	.79
H8b: pessi-opti	−0.218	.48	−0.652	.05	−0.522	.11	−0.585	.12	−0.448	.13

**Table 4:** Results of robustness tests, active users evaluated under different thresholds.

Configuration	Exponential random graph model parameters and *P* values													
	Base test, threshold=25.00 (%)		Robustness test 1, threshold=20.00 (%)			Robustness test 2, threshold=23.00 (%)			Robustness test 3, threshold=27.00 (%)			Robustness test 4, threshold=30.00 (%)		
	Coefficient	*P* value		Coefficient	*P* value		Coefficient	*P* value		Coefficient	*P* value		Coefficient	*P* value
H1: reciprocity	3.850	<.001		3.748	<.001		4.052	<.001		3.881	<.001		3.943	<.001
H2: type	0.240	<.001		0.243	<.001		0.259	<.001		0.260	<.001		0.271	<.001
H3: friend	3.473	<.001		3.071	<.001		3.635	<.001		3.293	<.001		3.130	<.001
H5: active_user	0.012	.76		−.067	.14		0.040	.31		0.181	<.001		0.232	<.001
H6: new_user	0.409	<.001		0.431	<.001		0.423	<.001		0.440	<.001		0.497	<.001
H7: optimistic	0.289	<.001		0.315	<.001		0.310	<.001		0.353	<.001		0.343	<.001
H7: pessimistic	0.144	.05		0.156	.04		0.177	.02		0.125	.04		0.168	.04
H8a: opti-opti	−0.332	.23		−0.069	.82		0.047	.82		−0.229	.36		−0.286	.24
H8a: pessi-pessi	−0.140	.71		−0.212	.54		−0.366	.31		−0.250	.37		−0.394	.20
H8b: opti-pessi	0.168	.51		0.204	.39		0.244	.37		0.166	.49		0.357	.30
H8b: pessi-opti	−0.218	.48		−0.354	.28		−0.591	.12		−0.529	.09		−0.400	.30

## Discussion

### Summary of Results

This study uses ERGM to explore patterns of information exchange and social support in web-based health communities. Table 5 summarizes the hypotheses testing results. For hypotheses that were not supported or only partially supported, additional implications were provided. Overall, we found that reciprocity could promote information exchanges effectively. When sharing health knowledge, the homophily effect was not strong in web-based health communities, and conversations were more likely to occur between users of different types (eg, patient and doctor, web service staff, and regular users). Web-based friends were very likely to exchange information frequently with each other. Newly registered users were overall associated with better chances of receiving replies from peers. Sentiment plays an important role in web-based health communities, and users with polarized sentiment tend to receive more replies. In particular, pessimistic users were associated with better chances of informational support when knowledge is exchanged. Most of such support came from optimistic users.

**Table 5:** Summary of research hypotheses and results.

Hypothesis	Result	Implications
Hypothesis 1: Reciprocated information exchange is likely to develop in web-based health communities.	Supported	In web-based communities, norm of reciprocity exists.
Hypothesis 2: Reciprocated information exchange is likely to develop between users who share similar concerns in web-based health communities.	Partially supported	In web-based communities, homophily effects are not strong when health information is exchanged.
Hypothesis 3: Reciprocated information exchange is likely to develop between users who are web-based friends.	Supported	In web-based communities, friends are likely to exchange messages often.
Hypothesis 4: Patients who receive social support tend to provide support to others who are not necessarily the support provider.	Not supported	Indirect reciprocity hardly exists in Web-based Health Community.
Hypothesis 5: Highly active users are more likely to receive replies as social support.	Not supported	Preferential attachment was found to be in the opposite direction in knowledge sharing communities.
Hypothesis 6: In web-based health communities, new users are more likely to receive replies as social support.	Partially supported	Users who recently registered tend to participate in discussions in multiple subforums rather than staying in one specific subforum.
Hypothesis 7: In web-based health communities, patients with polarized sentiment are more likely to receive replies on their posts.	Partially supported	Negative sentiment was found to have a unique promoting impact when seeking informational support in web-based health communities.
Hypothesis 8a: Patients are likely to receive replies from peers with similar sentiment valence. Hypothesis 8b: Patients are likely to receive replies from peers with opposite sentiment valence.	Partially supported	Communication between sentiment polarized patients has a complex pattern: only when information exchange is involved, optimistic users are more likely to give support to other sentiment polarized users.

### Contributions

Our research makes several contributions to the literature. First, this study made the first attempt to test the network exchange framework on reply networks developed in web-based health communities. Web-based health discussions are distinct from other types of conversations in that they contain sensitive and private information and specialized knowledge. Handling such information gives rise to special community norms that could lead to unique social exchange patterns [9,24]. Our study applied ERGM under a network exchange framework and identified a number of such unique patterns. This study complements the literature on network exchange theories and contributes to a better understanding of social exchange patterns in the web-based health community context. Specifically, compared with conventional social networking sites where the formation of social ties is driven by homophily effects, we found that conversations between users of different types were more common when users discussed diabetes knowledge. It does not conflict with prior findings in the network exchange framework because information exchange is different from simply making friends. User heterogeneity could actually increase the effectiveness of knowledge sharing [7]. In terms of preferential attachment, we found that the sentiment of users interacts with discussion topics during the formation of reply networks. Generally, showing polarized sentiment resulted in better chances of receiving replies. However, when seeking knowledge regarding disease, expressing negative emotion could be a better strategy. We further found that most users who provided social support to such users were optimistic users.

Second, our research used sentiment analysis to identify optimistic users and pessimistic users from web-based health communities. To the best of our knowledge, our study is the first to examine how users with different sentiments participate differently in information exchange and social support activities.

Practically, findings from this study help patients in web-based health communities to obtain information and social support more effectively. For example, in addition to making friends, patients are encouraged to participate in discussions on health care knowledge as well as personal life to increase their visibility in the community. It is fine to express negative sentiment when seeking informational support, and showing a positive attitude could be more helpful when making friends with others.

### Limitations

A limitation of this study is that our empirical analysis focused on a diabetes-related health community. Although we expect that similar patterns of information exchange and social support should be observed in other web-based health communities that provide web forums, it is interesting to see if the addition of other social features (eg, health platforms that provide feeds to users based on collaborative filtering) will affect how patients interact with each other. Moreover, the internet has changed dramatically over the 10-year time frame covered in this study. As the number of users increases over time, the resulting users in this study after network dichotomization might represent a more recent sample. Performing a temporal analysis to examine how social support patterns evolve dynamically could also be a future direction.

## Appendix

Multimedia Appendix 1 Research hypotheses, levels of analysis, and graphical illustrations.

## Abbreviations

ERGM: exponential random graph model
