# Supplementary figures and images for: Examining Patterns of Information Exchange and Social Support in a Web-Based Health Community: Exponential Random Graph Models
Source: J Med Internet Res. 2020 Sep 29;22(9):e18062. doi: 10.2196/18062 (PMC7556372; doi:10.2196/18062)

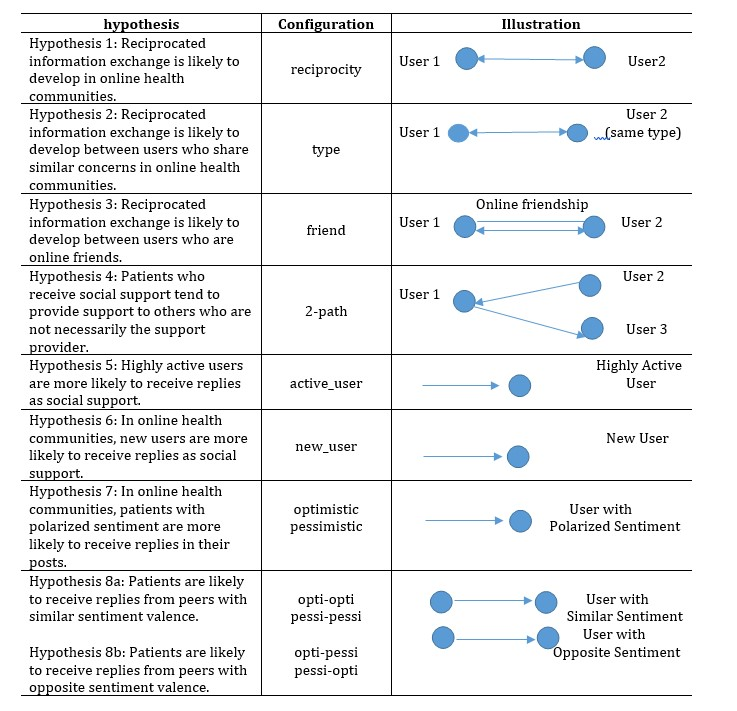

Supplement: Multimedia Appendix 1 [file jmir_v22i9e18062_app1.png]
